# Supplementary material for: Bone-in-culture array as a platform to model early-stage bone metastases and discover anti-metastasis therapies
Source: Nat Commun. 2017 Apr 21;8:15045. doi: 10.1038/ncomms15045 (PMC5413944; doi:10.1038/ncomms15045)
Supplement: Supplementary Information — Supplementary Figures and Supplementary Tables [file ncomms15045-s1.pdf]

Supplementary Table 1: Names and grouping information of the epigenomic modulators used in multiple parallel test on BICA. The grouping was performed in the first round of test, whereas individual drugs from most effective groups were tested in the second round.

**Group by Target**	**Names of Modulators**					
**HDAC-1**	Vorinostat (SAHA, MK0683)	Valproic acid sodium salt (Sodium valproate)	Panobinostat (LBH589)	Trichostatin A (TSA)	Entinostat (MS-275)	Belinostat (PXD101)
**HDAC-2**	PCI-24781 (Abexinostat)	LAQ824 (Dacinostat)	Quisinostat (JNJ-26481585)	Mocetinostat (MGCD0103)	Droxinostat	MC1568
**JAK**	Ruxolitinib (INCB018424)	AZD1480	LY2784544	AZ 960	CYT387	NVP-BSK805 2HCl
**Aurora**	PHA-680632	MLN8054	ZM 447439	CCT129202	Danusertib (PHA-739358)	
**PARP**	Veliparib (ABT-888)	Olaparib (AZD2281, Ku-0059436)	Iniparib (BSI-201)	Rucaparib (AG-014699,PF-01367338)	INO-1001	AG-14361
**Epi-reader Domain**	CPI-203	PFI-1 (PF-6405761)	I-BET151 (GSK1210151A)	UNC1215	(+)-JQ1	I-BET-762
**Histone Methyltransferase**	Entacapone	EPZ5676	SGC 0946	3-Deazaneplanocin A (DZNeP)	EPZ-6438	MM-102
**DNA Methyltransferase**	Decitabine	Azacitidine	Zebularine	SGI-1027	Lomeguatrib	RG108
**Sirtuin**	Quercetin	SRT1720	EX 527 (Selisistat)	Sirtinol	Resveratrol	
**Pim**	SGI-1776 free base	CX-6258 HCl	AZD1208	SMI-4a	DMSO	
**Mixture 1**	FG-4592	2-Methoxyestradiol (2-MeOE2)	IOX2	OTX015	UNC669	
**Mixture 2**	Tranylcypromine (2-PCPA) HCl	GSK J4 HCl	IOX1	OG-L002	C646	Procainamide HCl

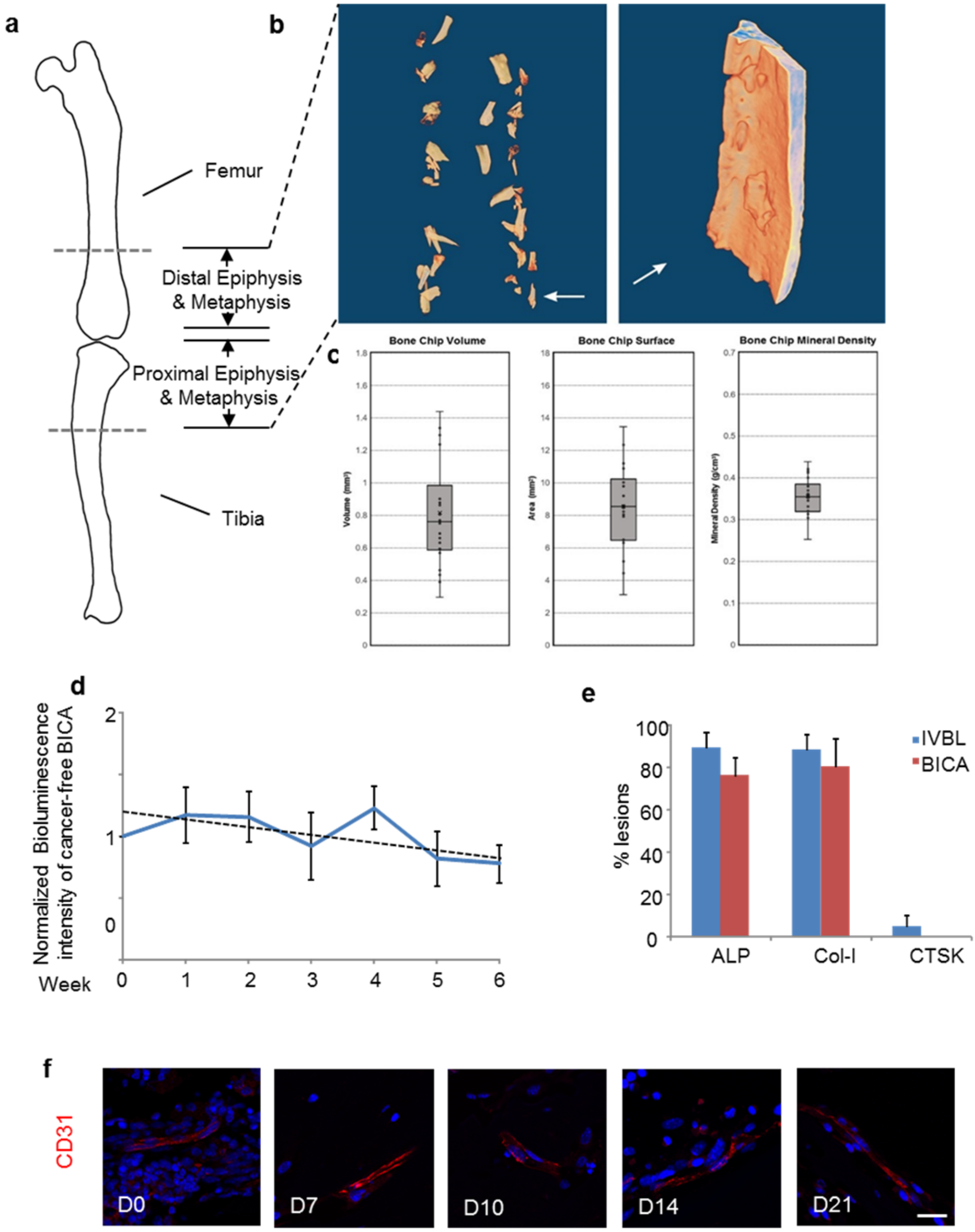

Supplementary Figure 1. BICA provides a bone-like microenvironment.

a. Illustration of the specific regions within hind limb bones that are enriched with disseminated MCF-7 cells after IIA injection and used for BICA.

b. 3D reconstruction of microCT for bone fragments used in BICA.

c. Distribution of bone fragment size (long diameter), surface area, and bone mineral density (BMD) in BICA. Error bars: S.D.

d. Bone tissues maintain viability in BICA condition for up to 6 weeks. Bone fragments from transgenic firefly luciferase mice were kept in tissue culture. Luciferase activities were measured weekly to track the viability of bone cells. N=6 bone fragments. Error bars: S.E.M.

e. Quantification of the staining of ALP, Col-I and CTSK in BICA and IVBL (in vivo bone lesions). Percent of lesions with at least two positively stained cells is shown. N=5 independent slides. Error bars: S.E.M.

f. Representative immunofluorescence images show that endothelial cells can survive in BICA for 3 weeks. Red- CD31+ cells. Scale bars: 25 µm.

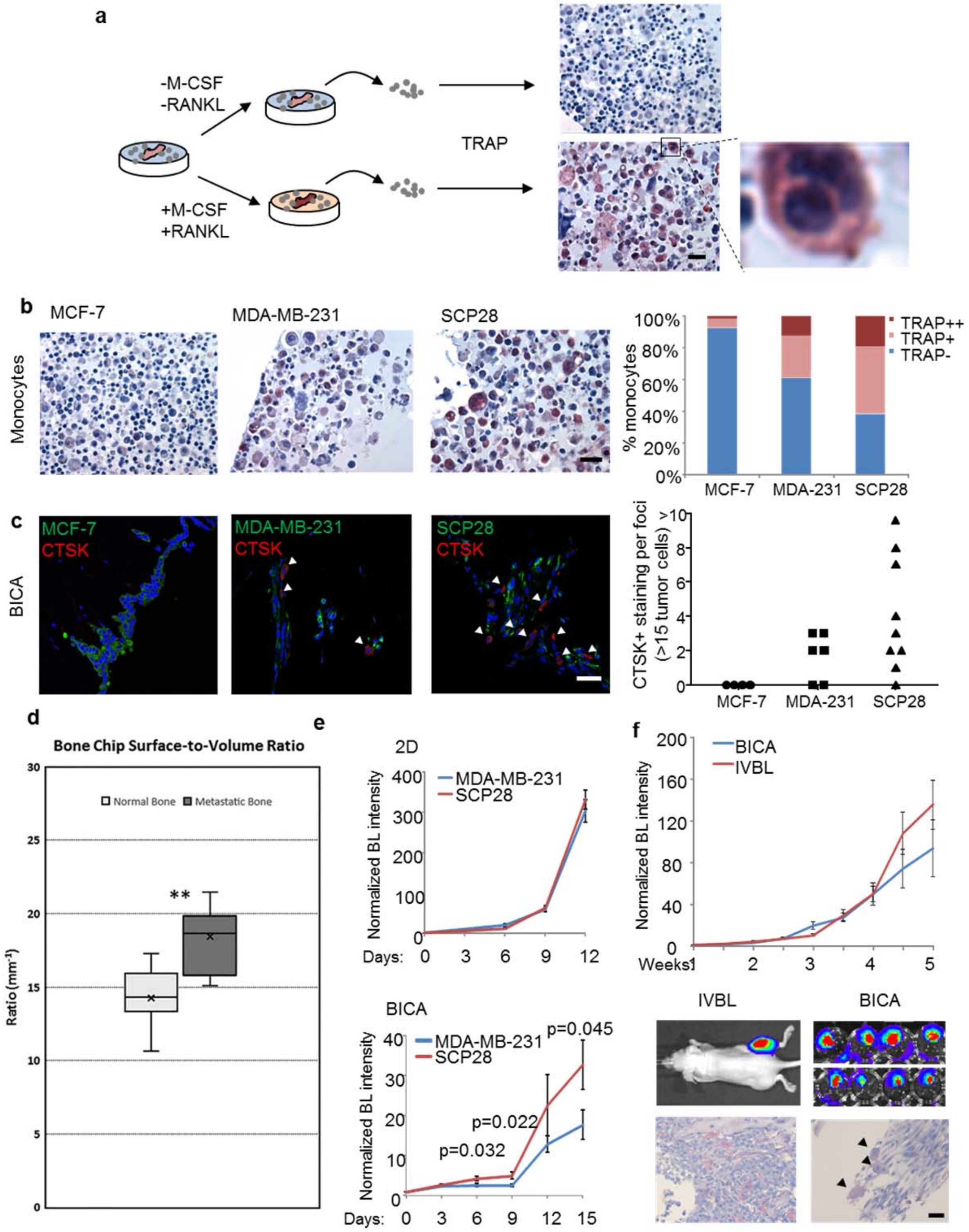

Supplementary Figure 2. BICA can be used to study advanced osteolytic metastasis.

a. M-CSF (20 ng/ml) and RANKL (100 ng/ml) are added to the hematopoietic cells in BICA. Ten days later, TRAP staining was performed to determine osteoclast activation. A TRAP+, multi-nuclear cell is shown. Scale Bar: 25 µm.

b. TRAP staining and quantification of BICA hematopoietic cells when MCF-7, MDA-MB-231 or SCP28 cells were used. No exogenous M-CSF or RANKL was added. Scale Bar: 25 µm.

c. CTSK staining and quantification of BICA samples bearing MCF-7, MDA-MB-231 or SCP28 cells after 3 weeks' culture in BICA. White arrows indicate the CTSK staining of activated osteoclasts. No exogenous M-CSF or RANKL was added. Scale Bar: 50 µm. Each dot represents a tumor foci containing >15 cancer cells.

d. Surface--to-volume ratio of BICA fragments with or without SCP28 lesions measured by microCT. Asterisk: $P<0.01$. P values are determined by *t*-test (2-tailed) on each time point. N=8 bone fragments for each group. Error bars: S.D.

e. Growth curves of MDA-MB-231 parental cells and the bone-tropic SCP28 cells in 2D cultures (upper) and in BICA (lower). P values are determined by *t*-test (2-tailed) on each time point. N=3 independent biological replicates for 2D cultures and 8 bone fragments for each group in BICA. Error bars: S.E.M.

f. Growth curves (upper) and representative Bioluminescence imaging (middle) or TRAP staining (lower) of SCP28 cell growth in BICA (N=16 bone fragments) and *in vivo* (N=4 mice) for 5 weeks. Metastatic burden was quantitated by BL intensity and normalized to week 1. Black arrows indicate the red TRAP staining of activated osteoclasts. No exogenous M-CSF or RANKL was added for BICA culture.

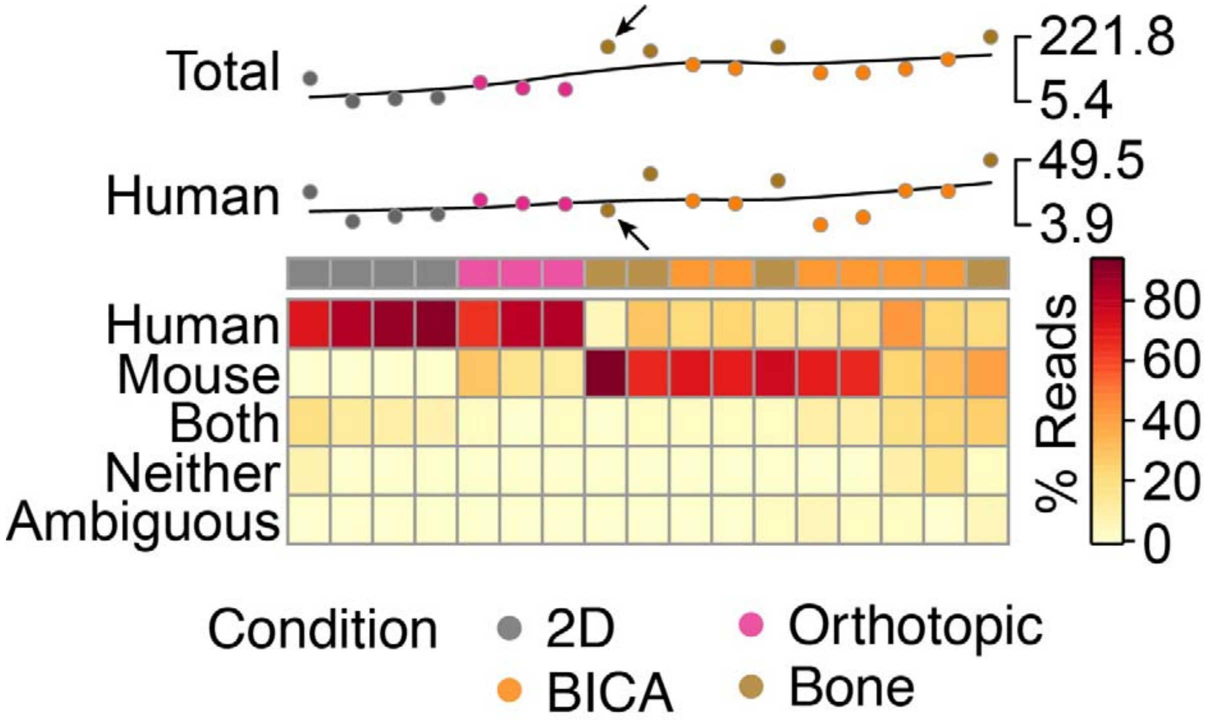

Supplementary Figure 3. Sequence mapping results of RNA-seq data.

Top: the number of total and human sequences in each specimen. Bottom: a heat map shows the percent reads that are successfully aligned to human or mouse reference genomes.

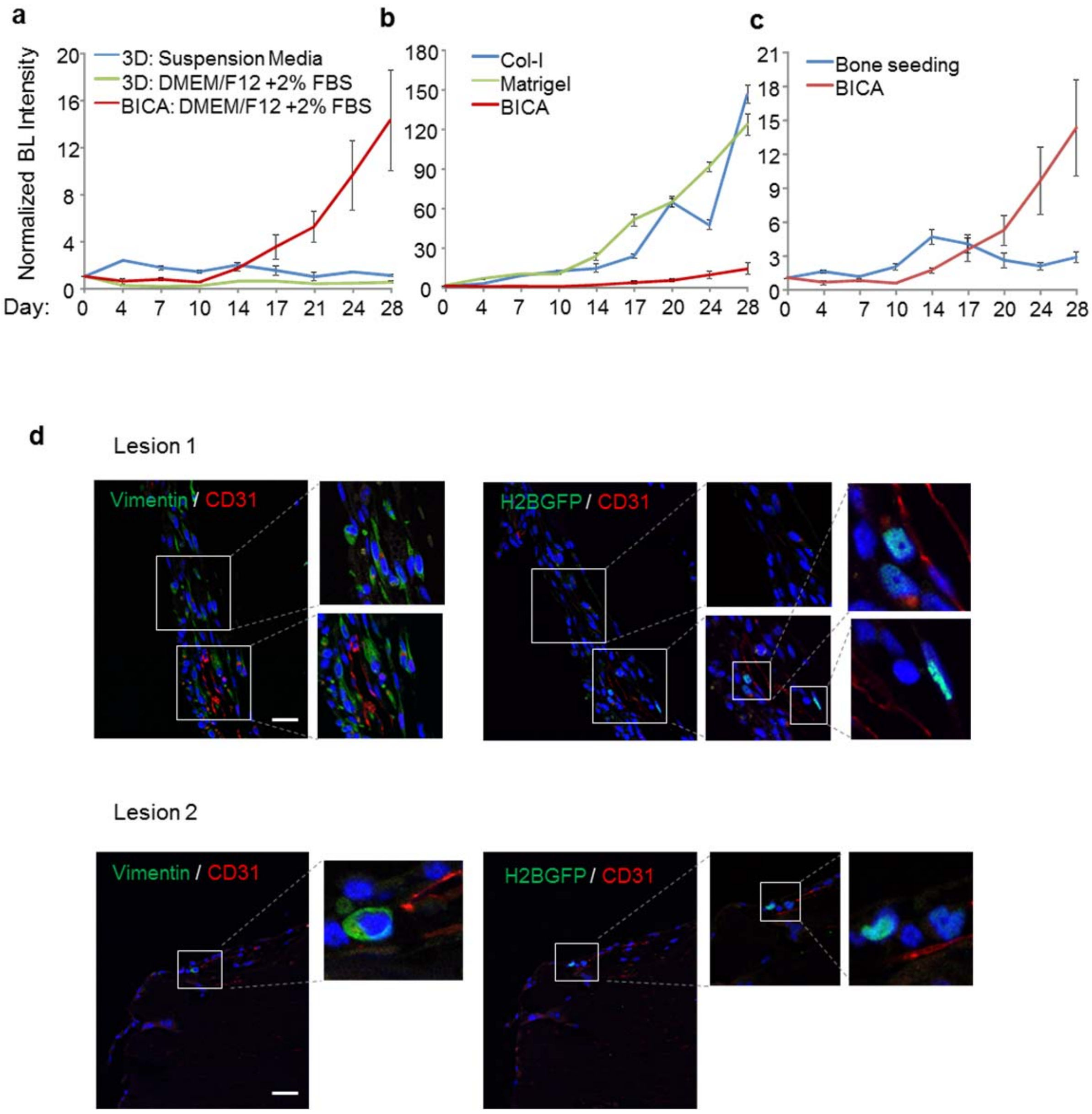
a
3D: Suspension Media
3D: DMEM/F12 +2% FBS
BICA: DMEM/F12 +2% FBS
Normalized BL Intensity
20
16
12
8
4
0
Day:
0 4 7 10 14 17 21 24 28
b
Col-I
Matrigel
BICA
180
150
120
90
60
30
0
0 4 7 10 14 17 20 24 28
c
Bone seeding
BICA
21
18
15
12
9
6
3
0
0 4 7 10 14 17 20 24 28
d
Lesion 1
Vimentin / CD31
H2BGFP / CD31
Lesion 2
Vimentin / CD31
H2BGFP / CD31

Supplementary Figure 4. Other ex vivo models do not recapitulate kinetics of bone colonization.

a-c. Kinetics of bioluminescence signals of MCF-7 cells that constitutively express Fluc/GFP in 3D cultures (**a**) (6 wells as technical replicates for each time point), ECM coating models (**b**) (wells pre-coated by either Matrigel or Collagen I, 12 wells as technical replicates), and ex vivo models utilizing natural bone tissues (**c**) (IIA injection of 5×10^5 cells, n=8 fragments for BICA; 2×10^4 cells/well, n=7 fragments for "Bone seeding"). Cancer cells are quantitated by bioluminescence (BL) intensity. All data are normalized to Day 0 values right after injection or seeding. Error bars: S.E.M. The same BICA curve was shown as reference in all 3 panels. The experiment was replicated twice in the laboratory with consistent results and a representative one is shown.

d. Representative image and quantification of dormant (H2B-GFP+), Vimentin+ MDA-MB-231 cells residing on microvascular endothelium in BICA culture. Lesion 1 shows cancer cells with/without microvascular endothelium surrounding. Consecutive sections were used for the same lesion. Red: CD31+ endothelial cells, green: H2B-GFP+ or Vimentin+ cancer cells. Scale bars: 50 µm.

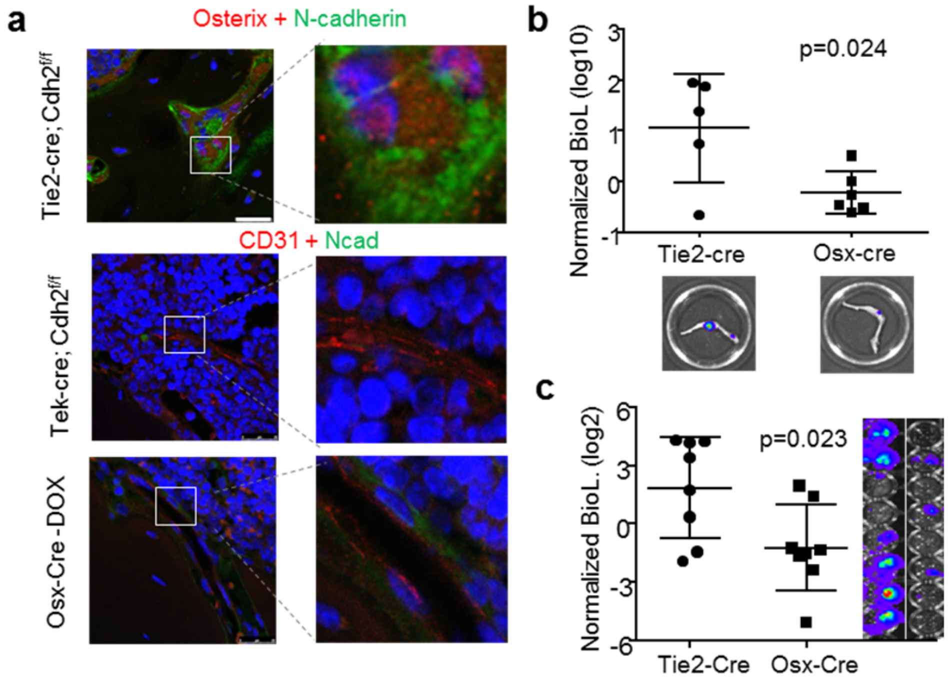

Supplementary Figure 5. BICA recapitulates cancer-niche interaction.

a. Representative fluorescence staining shows conditional knockout of N-cadherin in endothelial cells. Top row: red- osterix+ cells, green- N-cadherin. Middle and bottom rows: red- CD31+ cells, green- N-cadherin. Scale bars: 25 µm.

b. Conditional KO of N-cadherin in Osterix+ cells results in significant decrease of bone colonization of AT-3 cells as compared to KO of N-cadherin in Tie2+ endothelial cells. N=5/6 animals for *Tie2-cre* and *Osx-cre* group, respectively. Error bars: S.D. The experiment was replicated twice and showed consistent results. Data shown is the combined results.

c. Conditional KO of N-cadherin in Osterix+ cells results in significant decrease of tumor growth of MCF-7 cells in BICA as compared to KO of N-cadherin in Tie2+ endothelial cells. N=8 bone fragments. Error bars S.D.

P values are determined by Student's *t*-test (2-tailed).

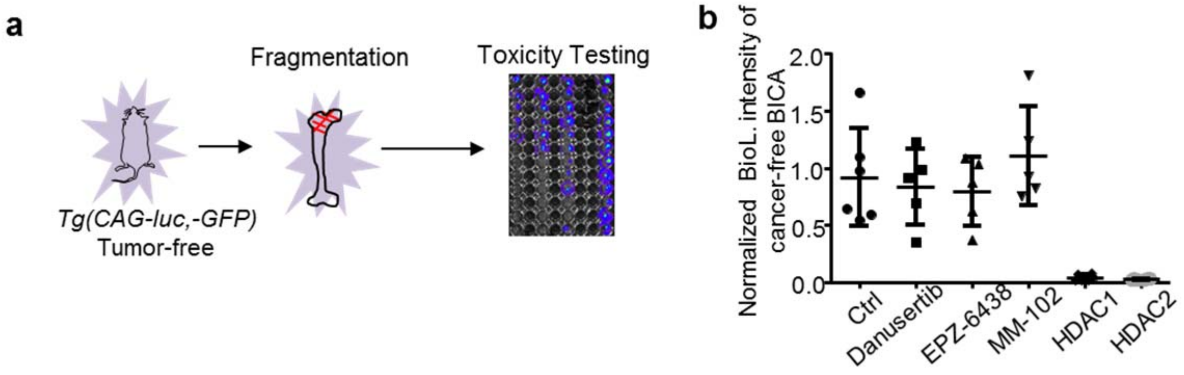

Supplementary Figure 6. BICA can be used for toxicity tests.

a. Schematic shows toxicity test using transgenic luciferase mice. Cancer-free bones expressing firefly luciferase are fragmented and treated by the same drugs used for BICA.

b. Drugs mentioned in the main text are used to treat cancer-free bone tissues to quantitate their toxicity on normal bone tissues. N=6 for Ctrl, HDAC1 and HDAC2 group; N=5 for Danusertib, EPZ-6438 and MM-102 group. Error bars: S.D.

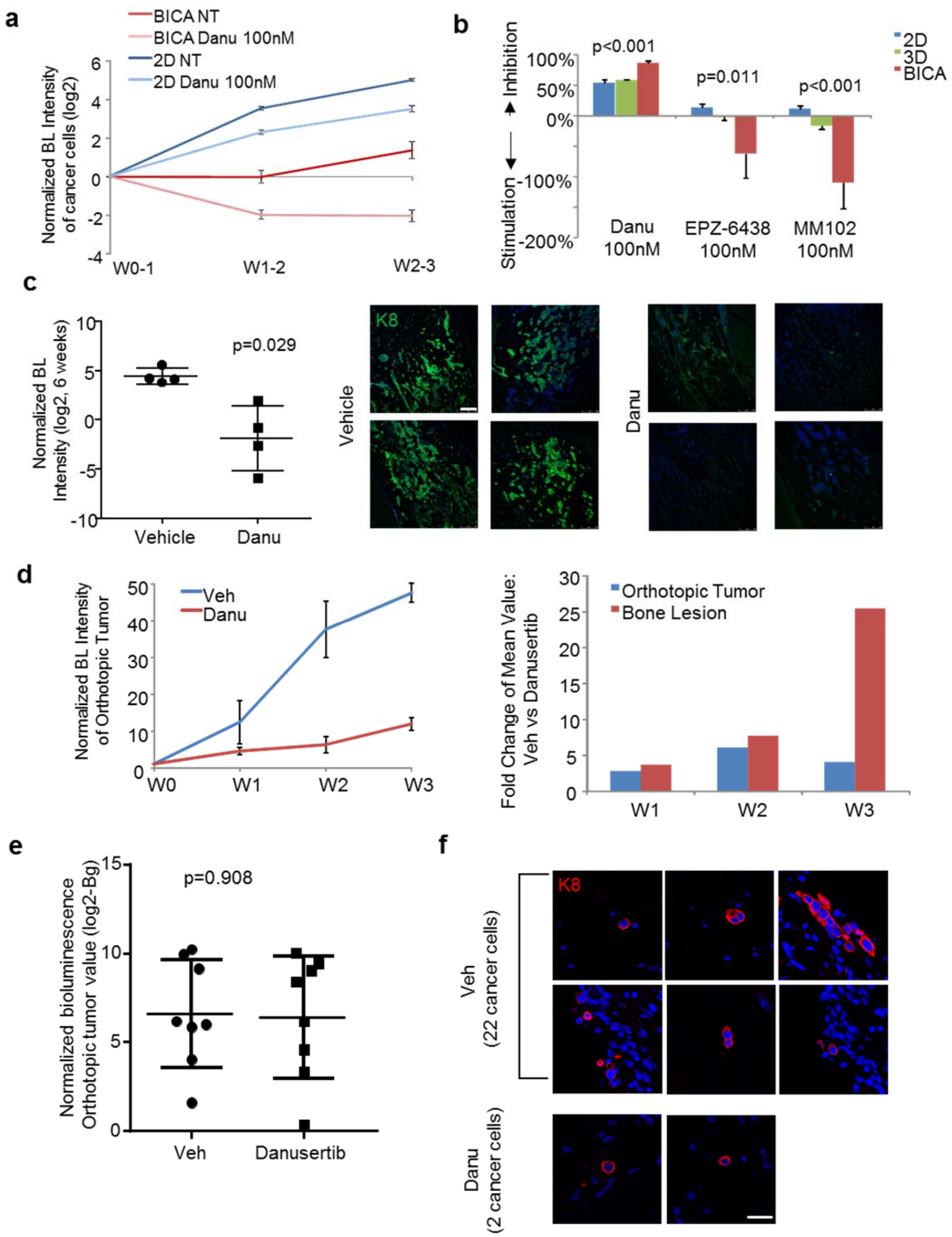

Supplementary Figure 7. BICA as a pre-clinical platform for rapid tests of anti-bone metastasis drugs.

a. Growth kinetics of MCF-7 with and without 100nM Danusertib in both 2D and BICA culture. Log-transformation was performed to better display the relative effect size of danusertib in different settings. Error bars: S.E.M.

b. Inhibition rate of MCF-7 cells by 100nM danusertib, EPZ-6438 and MM-102 in 2D, 3D or BICA culture. For 2D and 3D cultures, six technical replicates were included in each group. For BICA, N=6 bone fragments for each condition. Samples are under corresponding treatment for 3 weeks and measured by bioluminescence. P values are determined by ANOVA tests.

c. An experiment analogous to Fig. 5d, showing the tumor burden (K8+ MCF-7) measured by bioluminescence intensity (left) and fluorescence signals (right) with/without Danusertib treatment for 6 weeks. Error bars: S.D. Scale bar: 250 µm.

d. The effects of Danusertib on fat pad-injected MCF-7 orthotopic tumors. Left: growth curves as measured by in vivo BL imaging. N=5 mice per group. P values are determined by repeated measures ANOVA tests on the growth curves. Error bars: S.E.M. Right: Comparison of tumor burden fold change of Vehicle vs. Danusertib treatment between orthotopic tumors and bone lesions.

e. The effects of danusertib on recurrent orthotopic tumors in 4T1.2 model. The treatment of danusertib started after orthotopic tumor resectioning. Signal intensity was quantitated 12 days after the surgery, and was quantitated as shown in the dot plot. N=8 animals in each experimental group. The P value was determined by *t*-test (2-sided). Error bars: S.D

f. Immunofluorescence staining of the MCF-7 cells (K8-positive, red) in BICA section after two weeks' treatment after IIA injection. N= 8 bone fragments in each group. Scale bar: 25 µm.
